# Photosensing and Thermosensing by Phytochrome B Require Both Proximal and Distal Allosteric Features within the Dimeric Photoreceptor

**DOI:** 10.1038/s41598-017-14037-0

**Published:** 2017-10-20

**Authors:** E. Sethe Burgie, Adam N. Bussell, Shu-Hui Lye, Tong Wang, Weiming Hu, Katrice E. McLoughlin, Erin L. Weber, Huilin Li, Richard D. Vierstra

**Affiliations:** 1 Department of Biology, Washington University in St. Louis, St. Louis, Missouri 63130 USA; 2 Department of Genetics, University of Wisconsin-Madison, Madison, Wisconsin 53706 USA; 3 Department of Biology, Brookhaven National Laboratory, Upton, New York, 11973 USA; 4 Van Andel Research Institute, Grand Rapids, Michigan 49503 USA; 5 CUNY Advanced Science Research Center, The City University of New York, New York, New York 10031 USA

## Abstract

Phytochromes (Phys) encompass a diverse collection of bilin-containing photoreceptors that help plants and microorganisms perceive light through photointerconversion between red light (Pr) and far-red light (Pfr)-absorbing states. In addition, Pfr reverts thermally back to Pr via a highly enthalpic process that enables temperature sensation in plants and possibly other organisms. Through domain analysis of the Arabidopsis PhyB isoform assembled recombinantly, coupled with measurements of solution size, photoconversion, and thermal reversion, we identified both proximal and distal features that influence all three metrics. Included are the downstream C-terminal histidine kinase-related domain known to promote dimerization and a conserved patch just upstream of an N-terminal Period/Arnt/Sim (PAS) domain, which upon removal dramatically accelerates thermal reversion. We also discovered that the nature of the bilin strongly influences Pfr stability. Whereas incorporation of the native bilin phytochromobilin into PhyB confers robust Pfr → Pr thermal reversion, that assembled with the cyanobacterial version phycocyanobilin, often used for optogenetics, has a dramatically stabilized Pfr state. Taken together, we conclude that Pfr acquisition and stability are impacted by a collection of opposing allosteric features that inhibit or promote photoconversion and reversion of Pfr back to Pr, thus allowing Phys to dynamically measure light, temperature, and possibly time.

## Introduction

The biological perception of light is mediated by a collection of photoreceptors that connect light absorption to various signal transduction cascades crucial to organismal development and survival. Influential photoreceptors among plants, bacteria, and fungi are the phytochromes (Phys), a superfamily of chromoproteins that enables light detection through a covalently-linked bilin (or linear tetrapyrrole) prosthetic group^1,2^. Phys typically exist in two conformers, a dark-adapted, red light-absorbing Pr state that is biologically inactive, and a metastable, far-red light-absorbing Pfr state that is biologically active. Through the reversible photointerconversion of Pr and Pfr and thermal reversion of Pfr back to Pr, Phys act as photoswitches in various signaling pathways that detect light fluence rate, duration, and spectral quality. Moreover, at least some plant Phys also perceive temperature through enthalpic effects on the rate of thermal reversion^3,4^, and can possibly measure photoperiod via the nighttime depletion of Pfr^5^. The cumulative effects of Pr/Pfr interconversion impact a number of physiological processes, including photoprotection, redox sensing, phototaxis, sporulation, pathogen virulence and chromatic adaptation in microorganisms, and seed germination, chloroplast development, shade avoidance, circadian entrainment, flowering time and senescence in plants^6–8^.

Phys are head-to-head homodimers with each polypeptide harboring an N-terminal photosensory module (PSM) that binds the bilin followed by a C-terminal output module (OPM) that supports dimerization and often promotes signal transmission to downstream effectors^1,8^. The canonical PSM sequentially includes a variable N-terminal extension (NTE), Per/Arnt/Sim (PAS) and cGMP phosphodiesterase/adenylyl cyclase/FhlA (GAF) domains that form a tight complex through a unique figure-of-eight knot, and a Phy-specific (PHY) domain that is integral to Pfr acquisition and transmission of the light signal to the OPM. Whereas proteobacterial and fungal Phys link the bilin biliverdin IXα (BV) via the C3^2^ position of the A pyrrole ring vinyl sidechain and a conserved cysteine in the NTE^9^, cyanobacterial and plant Phys link the bilins phycocyanobilin (PCB) and phytochromobilin (PΦB), respectively, via C3^1^ of the A pyrrole ring ethylidine side chain and a conserved cysteine in the GAF domain^10,11^. Regardless of the linkage site, the incorporated chromophores assume near equivalent positions within the GAF domain cleft, where an intricate network of non-covalent contacts  enables the unique reversible absorption properties of Phys^1^. The OPM varies within the superfamily; it is often a histidine kinase domain in microbial Phys, and a histidine kinase-related domain (HKRD) with possibly other activities in the plant versions^1,8,12^.

Through a variety of structural and biophysical studies, a model for Phy photoconversion is emerging^1,13–16^. Following photoexcitation of Pr, the bilin reversibly switches from a *15Z* to *15E* configuration. The net effect is a ~180**°** rotation of the D pyrrole ring, followed by sliding the bilin within the GAF domain pocket and reorientation of adjacent amino acids^14,17,18^. Subsequently, a structurally unique hairpin loop (also tongue or arm), extending from the PHY domain to contact the GAF domain surface near the bilin, transitions from a two-stranded, antiparallel β-sheet as Pr to a helical conformation as Pfr. Accumulating evidence posits that this reconfiguration rearranges the hairpin/GAF domain contact, which then tugs on the PHY domain. The end result is to effectively toggle the position/activity of the sister OPMs rigidly tethered to the PSM through a long helical spine^14,16,19,20^, but the end conformation remains unclear at present.

Given how sensitive Phy signaling is to Pr/Pfr interconversion, it is not surprising that multiple features both proximal and distal to the bilin are crucial to facilitate or modify this exchange. This is especially evident for plant Phys, some of which must be delicately balanced to perceive both light and temperature^3,4^. Within the GAF domain pocket, a number of amino acids are essential for acquisition of Pfr, with substitutions of key bilin/protein contacts either blocking the Pr → Pfr transition or stabilizing/destabilizing Pfr once formed^14,15,21–24^. In fact, million-fold differences in the rate of thermal reversion are possible by substituting individual residues in this pocket, without significant impact on absorption or photoconversion^21^.

Outside of the GAF domain, additional features also strongly influence thermal reversion, suggesting that the overall conformation of the Phy dimer allosterically impacts Pfr stability even at considerable distances away from the bilin^19,21,25^. For example, manipulating the contact points between the hairpin and GAF domain either substantially promotes or dampens Pfr → Pr thermal reversion^21,26,27^, whereas the NTE in plant Phys is important for stabilizing the Pfr state and subsequent signaling once formed^21,28–30^. Interestingly, the effects of the NTE on thermal reversion can also be reversibly adjusted by phosphorylation *in planta*, thus suggesting that controls on Pfr stability by this region are physiologically relevant^28,31,32^. Even the OPM impacts Pfr stability. For example, removal of the OPM histidine kinase domain in the *Deinococcus radiodurans* bacteriophytochrome (BphP) greatly dampens thermal reversion despite being largely separated from the GAF domain in the 3-D dimeric structure of the photoreceptor^14,19,25,33^.

To further define how various features within the dimeric Phy molecule impact Pr/Pfr interconversion, we analyzed the quaternary and spectroscopic properties of a collection of mutations within *Arabidopsis thaliana* PhyB, a prominent isoform crucial for both light and thermal perception in plants^3,4,7,26^, and now widely used for the optogenetic control of cellular events (e.g.,^34,35^). PhyB-type isoforms harbor the canonical PAS-GAF-PHY modular architecture of the PSM but are uniquely preceded by a long glycine/serine-rich NTE^21,36^. Like other plant Phys, the PSM of Arabidopsis PhyB is followed by a PAS-PAS and HKRD region that helps promote dimerization, nuclear import, and possibly signaling through interaction with a family of PHYTOCHROME-INTERACTING FACTOR (PIF) transcriptional repressors^21,37^.

Remarkably, we found that several features, both proximal to the bilin and anticipated to be substantially distant within the 3-D structure, have strong effects on Pfr acquisition and stability, and thus critical to proper light and thermal perception. Even subtle substitution of the native chromophore PΦB for PCB strongly dampens thermal reversion. Defining how these modular features evolved to modify photo- and thermosensing of the various Phy isotypes should provide a framework to reengineer these photoreceptors for agronomic benefit and as improved optogenetic reagents.

## Results

### Domains within the OPM promote PhyB dimerization

**Figure 1:**
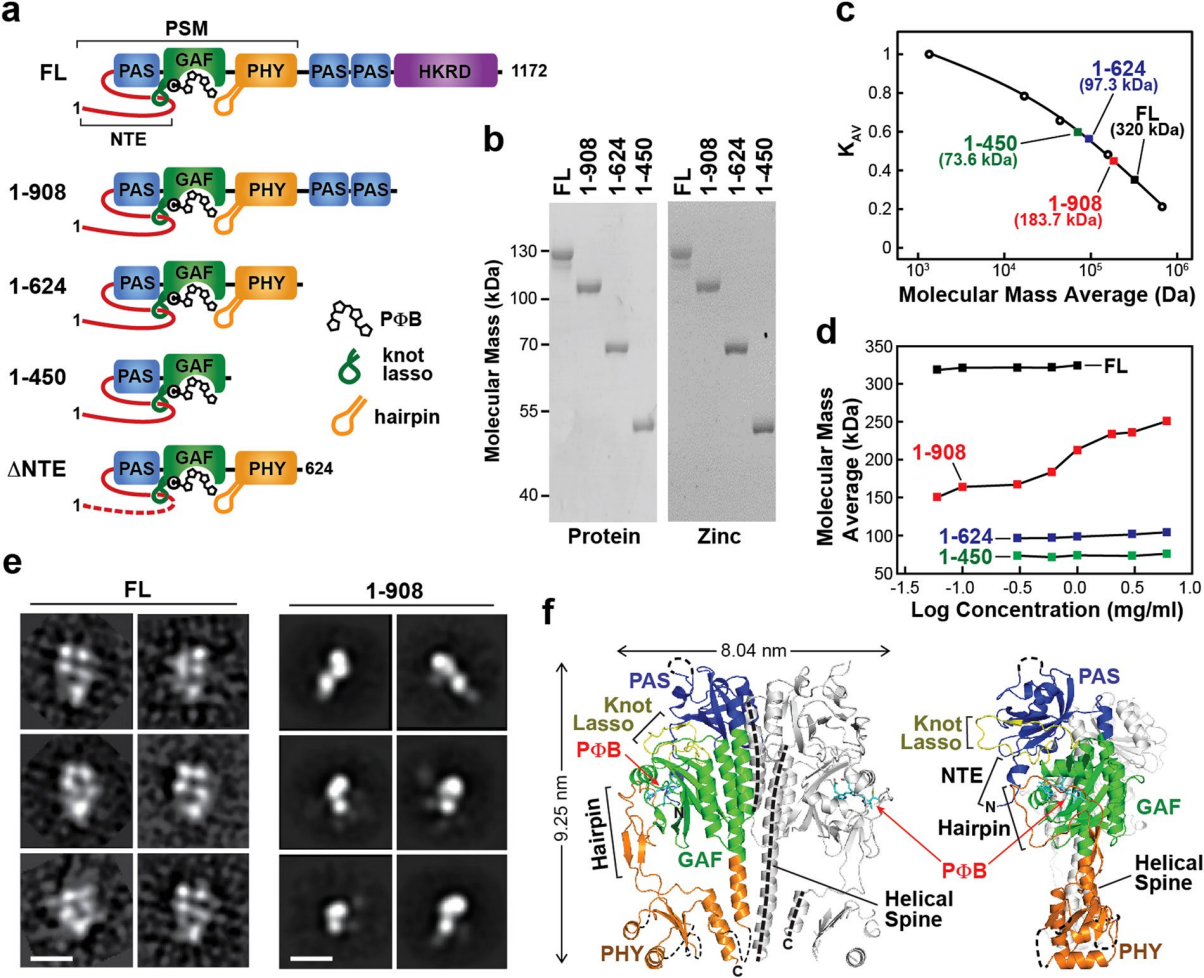
The OPM is required for oligomeric assembly of PhyB. **(a)** Domain organization of Arabidopsis PhyB and composition of the truncation mutants used in this study. The numbers of the N- and C-terminal residues are indicated. PAS, GAF, PHY and HKRD segments are labeled. The bilin, figure-of-eight knot, and hairpin features are indicated. C represents the cysteine that covalently binds PΦB. **(b)** Assembly of the samples with PΦB. Preparations were subjected to SDS-PAGE and either stained for protein with Coomassie Blue or for the bound bilin by zinc-induced fluorescence. For full-length images of the SDS-PAGE gels see Supplementary Fig. S1. **(c)** Quaternary mass of FL PhyB and truncations as determined by SEC of the preparations at 0.6 mg/ml concentration. The calculated masses based on a suite of size standards are indicated. K_AV_ = (V_e_ − V_0_)⋅(V_t_ − V_0_)^−1^, where K_AV_ is the molecular partition function, V_e_ is the elution volume, V_0_ is the void volume as measured by blue dextran, and V_t_ is the volume accessible to solvent as measured by vitamin B_12_. **(d)** The size dependence of FL PhyB and the truncations on chromoprotein concentration. **(e)** Single-particle EM images of FL PhyB and the 1–908 truncation generated after negative staining. Selected reference-free class averages assembled from the electron micrographs are shown. The scale bar indicates 10 nm. **(f)** Orthogonal ribbon views of the crystal structure of dimeric PhyB(90–624) fragment (PDB: 4OUR) for comparison to the EM images in panel (e). The molecular dimensions of the model are indicated. The PAS, GAF, and PHY domains are colored in blue, green and orange, respectively. PΦB is in cyan. The helical spine, hairpin, knot lasso, and NTE features are highlighted. N, N-terminus. C, C-terminus.

**Figure 2:**
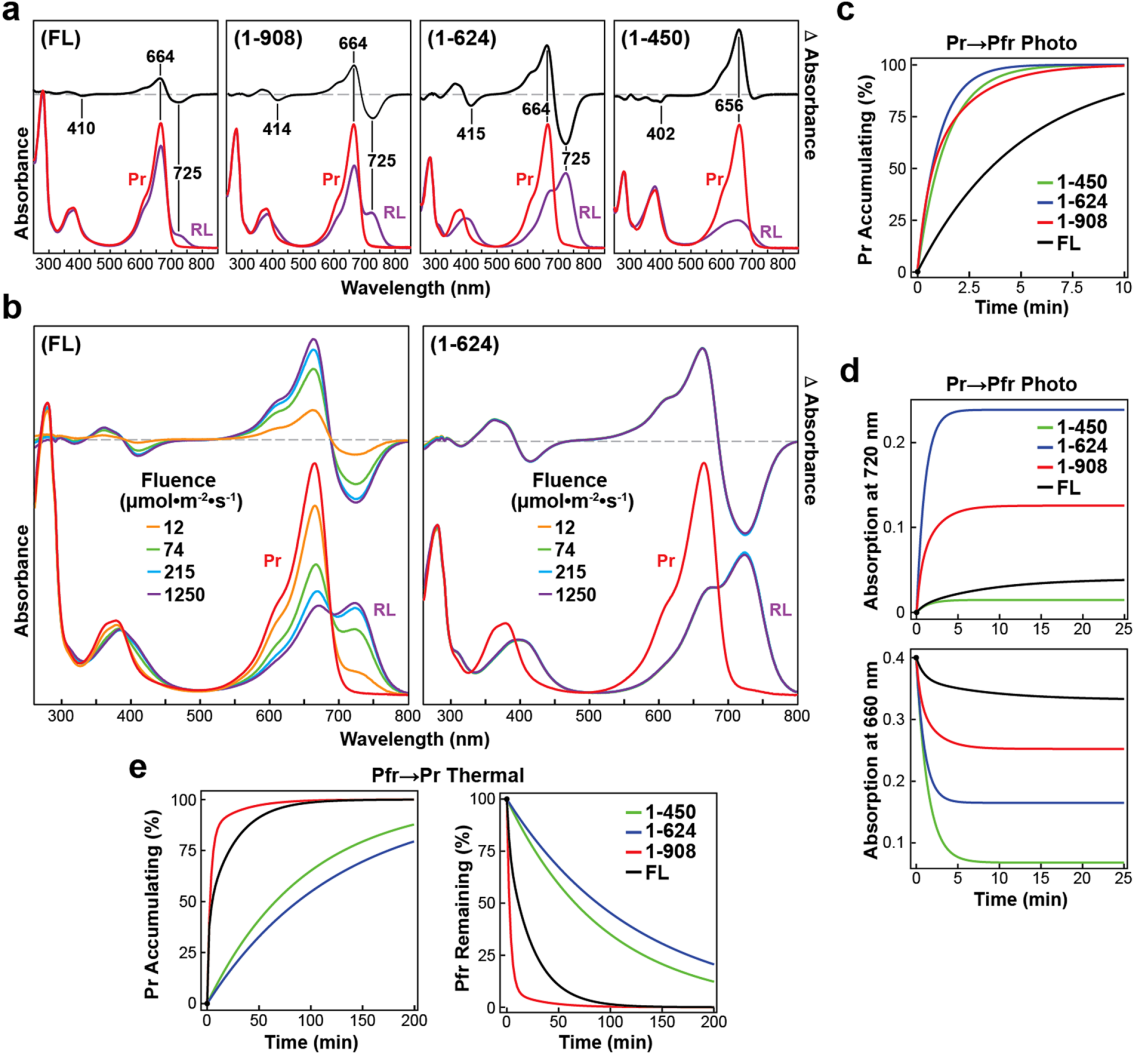
C-terminal truncations of PhyB alter its spectroscopic and kinetic properties. **(a)** UV-Vis absorption and difference spectra for full-length (FL) PhyB and various C-terminal truncation mutants assembled with PΦB. Spectra were recorded after dark-adaption (Pr, red) or following irradiation with 12 μmol⋅m^−2^⋅s^−1^ red light until steady state was reached (RL, purple). The absorption and difference maxima/minima are indicated. The difference spectra are shown at 70% of the total amplitude. Domain organization of the C-terminal truncations assembled are described in Fig. 1a. (**b**) UV-Vis absorption and difference spectra of Pr samples exposed to increasing fluence rates of 660-nm red light. **(c)** Normalized kinetic profiles of Pr → Pfr photoconversion for samples irradiated with 660 nm light at 12 μmol⋅m^−2^⋅s^−1^ as monitored at 720 nm. Examples of the raw kinetic traces can be found in Supplementary Fig. S2. **(d)** Simulated kinetic profiles for photoconversion as monitored by the gain in Pfr absorption at 720 nm (top) or loss of Pr absorption at 660 nm (bottom) to show the relative absorption changes for the samples. **(e)** Pfr → Pr thermal reversion of the samples as monitored by the gain in Pr absorption at 660 nm (top) and loss of Pfr absorption at 720 nm (bottom). Q band absorption maxima was 0.4 for Pr spectra of (**a**,**b**). Kinetic traces found in (**c**–**e**) were calculated from rate constants listed in Table 1.

To provide further insights into how various modular features within Phys influence their dimeric structure and Pr/Pfr interconversion, we first studied a series of C-terminal truncation mutants (1–908, 1–624, and 1–450) of Arabidopsis PhyB that sequentially eliminated the HKRD, the pair of internal PAS domains, and the PHY domain, respectively, from the full-length 1172-residue bili-protein (Fig. 1a). The 1–908 fragment was of particular interest given its widespread use as an optogenetic reagent in combination with its strong binding partners PIF3/6^34,35,38^. Analysis of the GAF domain by itself was not attempted given that its intimate connection to the N-terminal PAS domain through the figure-of-eight knot appears to be required for proper folding^21^. The truncations were expressed and assembled with PΦB in *Escherichia coli*, purified via appended tags bearing a hexahistidine (6His) moiety followed by a tobacco etched virus (TEV) protease cleavage site, and separated from the tag by TEV protease digestion. As shown in Figs 1 and 2, purified chromoproteins were readily obtained with high bilin occupancy and near normal Pr absorption, indicating that none of the missing features impacted the bilin lyase activity intrinsic to the GAF domain or substantially perturbed the correct association of PΦB in the Pr binding pocket. The largest variation was found for the 1–450 fragment, which displayed an 8-nm hypsochromic shift in the red Q-band absorption peak and a subtle change in the blue-light absorbing Soret peak as Pr (Fig. 2a).

Previous measurements of quaternary structure by equilibrium sedimentation and size exclusion chromatography (SEC) indicated that the 1–624 fragment of PhyB is monomeric in solution^21^, while the full-length chromoprotein should be dimeric based on prior analyses of the PhyA isoform^39,40^ and PhyB assembled with PCB in yeast^41^. Here, we confirmed by SEC that both the 1–450 and 1–624 fragments are monomers with observed masses of 73.6 and 97.3 kDa, respectively, which are consistent with the behavior of a non-spherical monomer, and that the full-length polypeptide is indeed a homodimer with an observed mass of 320 kDa (Fig. 1c). Similar analysis of the 1–908 fragment surprisingly provided an intermediate mass of 183.7 kDa, which agreed with the predicted mass of a spherical dimer but was in between the monomeric and dimeric forms in size if we assumed a non-spherical shape. Because Phys are likely to be non-spherical^15,20,33^, we hypothesized that the 1–908 fragment maintains a dynamic equilibrium between monomers and dimers. To test this possibility, we examined by SEC the dependence of solution size on protein concentration. Whereas full-length PhyB and the 1–450 and 1–624 species remained dimeric and monomeric, respectively, at all concentrations tested, the observed molecular mass of the 1–908 fragment strongly increased with protein concentration, implying mass-action conversion from monomeric to dimeric or higher-order oligomeric species (Fig. 1d).

To help support the finding that the 1–908 fragment behaves as a monomer at low protein concentrations, we developed single particle, negatively-stained EM images. Like previous studies with *D*. *radiodurans* BphP^15,33^, full-length Arabidopsis PhyB appears to assemble as a head-to-head dimer in solution with an obvious cavity separating the sister PSMs, which was seen as a quadrate constellation of densities form by the separate PAS/GAF and PHY domains (Fig. 1e). Much of this shape compared favorably with the crystallographic structure of the PhyB PSM when juxtaposed (Fig. 1e,f)^21^. The C-terminal OPMs imaged as a single element by EM, likely reflecting intertwining of the sister regions along with sufficient mobility to obscure some of their densities, as observed by similar EM analysis of *D*. *radiodurans* BphP^15,33^. By contrast, the 1–908 fragment imaged as a single three-lobed species without obvious two-fold symmetry or a central cavity, as would be expected for a monomeric PAS/GAF/PHY/PAS-PAS species (Fig. 1e). Taken together, these images support previous studies that PhyB has dimerization contacts both in the PAS-GAF region and the HKRD^21,39^, and that removal of the HKRD contact is sufficient to monomerize the chromoprotein at low protein concentrations.

### The OPM impacts PhyB photochemistry and thermal reversion

Whereas the Pr absorption of the 1–450, 1–624 and 1–908 fragments was remarkably similar, progressive truncations from full-length PhyB markedly impacted Pfr formation and its thermal stability. Here, the reactions were conducted at protein concentrations at or below 0.5 mg/ml when most of the 1–908 fragment should be monomeric (Fig. 1d). All but the 1–450 fragment generated near normal Pfr absorption upon prolonged excitation with red light (Fig. 2a,b). Instead the Q band absorption from the 1–450 fragment bleached without evolution of the far-red peak. This bleached state then fully decayed back to Pr in darkness, consistent with previous studies showing that the PHY domain strongly encourages the transition to Pfr and/or its maintenance^21,23,42^. Based on studies with other Phys^23,43^, the bleached species seen for the 1–450 fragment might represent the deprotonated meta-Rc intermediate that fails to decay to Pfr upon reprotonation without the PHY domain.

The surprising finding was that deletion of the HKRD improved the efficiency of Pr → Pfr photoconversion. This effect was first seen when moderate fluences of red light were used for photoconversion (12 μmol∙m^−2^∙s^−1^), which resulted in 50% and 90% transformation of Pr to Pfr for the 1–908 and 1–624 fragments, respectively, but only trace conversion to Pfr for full-length PhyB as judged by appearance of the Q-band peak at 730 nm (Fig. 2a). Only when we increased the fluence rate almost 100 fold (1250 μmol∙m^−2^∙s^−1^) could we obtain near saturating Pfr occupancy for the full-length chromoprotein (Fig. 2b). Detailed kinetic analysis showed that simply removing the HKRD increased Pr → Pfr photoconversion by ~ 8 fold (Fig. 2c, Supplementary Fig. S2; Table 1), indicating that this region and/or its impact on dimerization provides a substantial constraint to photoconversion of the bilin within the GAF domain. We note that the Pfr spectrum of full-length PhyB generated with this high fluence rate was not completely coincident with those obtained with the truncations at low fluence rates, implying that the HKRD also encouraged accumulation of a bleached state in red light (Fig. 2b). To support this possibility, thermal reversion recovery of the Pr Q-band included an extremely fast kinetic phase (rate constant ~8 sec^−1^), which was not detected by a comparable depletion of the Pfr absorption band (Fig. 2e). Interestingly, the emergence of a well-defined isosbestic point at 689 nm, when a range of fluences was compared, implied that accumulation of Pfr and this bleached state was proportional after steady state was reached.

**Table 1:** Spectral and Kinetic Constants of Truncation and Point Mutants of Arabidopsis PhyB.

Mutation^a^	Absorption maxima^b^ (nm)		Photoconversion rate^c,d,e^ (min^−1^)		Therm rev^c,d,f^ (x1000 min^−1^)
	Pr	Pfr	Pr ⇒ Pfr	Pfr ⇒ Pr	Pfr ⇒ Pr
***Domain Truncations***					
**PΦB**					
1–450	658	707	0.72^g^	0.11 (±0.02)	10.5 (±0.1)
1–624	664	724	0.98 (±0.03)	2.45 (±0.04)	7.91 (±0.02)
1–908^h^	665	724	k_1_ 2.1 (±0.3)	2.6 (±0.2)	320 (±20)
			k_2_ 0.47 (±0.05)		34 (±3)
FL^h,i^	664	725	k_1_ 1.1		410
			k_2_ 0.12		40
**PCB**					
1–450	643	693	1.04 (±0.05)	0.38 (±0.02)	38.4 ( ± 0.1)
1–624	651	714	1.35 (±0.04)	3.52 (±0.03)	0.52 (±0.01)
1–908	651	714	1.20 (±0.08)	3.52 (±0.06)	29.9 (±0.3)
***NTE Truncations***					
10–624	665	724	0.86 (±0.04)	2.43 (±0.01)	9.54 (±0.02)
20–624	665	724	0.82 (±0.09)	2.52 (±0.05)	10.1 (±0.1)
30–624	665	724	0.84 (±0.03)	2.54 (±0.07)	7.7 (±0.5)
41–624	665	725	0.88 (±0.03)	2.60 (±0.06)	6.50 (±0.02)
46–624	666	725	10.8 (±0.1)		
51–624	664	723	1.1 (±0.1)	2.53 (±0.06)	16.2 (±0.1)
55–624	665	724			14.9 (±0.1)
56–624	665	723			32.3 (±0.1)
57–624	665	722	0.97 (±0.05)	2.35 (±0.09)	50.1 (±0.1)
59–624	664	719			91.8 (±0.3)
60–624	665	720	1.09 (±0.02)	2.39 (±0.06)	85 (±1)
65–624	666	717	1.18 (±0.09)		143 (±1)
70–624	665	717	1.05 (±0.06)	2.28 (±0.03)	146 (±1)
80–624	664	717	1.17 (±0.03)	2.3 (±0.1)	169 (±1)
90–624	664	717	1.3^g^	2.5^g^	163 (±1)
100–624	664	717	0.99 (±0.05)	2.2 (±0.2)	134 (±1)
***PSM Point Mutants***					
S55A,K56A	666	725			16.3 (±0.1)
Q59A,Q60A	666	721			35.5 (±0.1)
Y61A,T62A	665	725			7.53 (±0.01)

^a^Data were collected from PhyB containing the indicated residues after removal of the N-terminal 6His-TEV tag.

^b^Maxima of the Q absorption band. Pfr maxima were identified from difference spectra generated by subtracting red-irradiated from dark-adapted samples.

^c^Calculated from the global fit of absorption at 640 nm, 650 nm, 660 nm, 670 nm, 700 nm, 710 nm, 720 nm, 730 nm, and 740 nm as a function of time using the equation: Abs = ΔAbs_total_•exp(−k•Time) + Abs_initial_.

^d^Values in parentheses represent the average of three technical replicates (±SD).

^e^Fluence rate was calibrated to 12 μmol•m^−2^•s^−1^ at the intersecting spectrophotometric light path.

^f^Fluence rate was calibrated to a nominal 0.465 μmol•m^−2^•s^−1^ for each measurement.

^g^Average of two technical replicates.

^h^Pr ⇒ Pfr photoconversion, Pfr ⇒ Pr photoconversion, and/or thermal reversion were expressed as the sum of two exponentials using the equation: Abs = ΔAbs_total1_•exp(−k_1_*Time) + ΔAbs_total2_•exp(−k_2_•Time) + Abs_initial_.

^i^Data were collected from a single sample.

When the C-terminal truncations were then analyzed for Pfr → Pr thermal reversion, a striking impact on Pfr stability was also seen (Fig. 2e). Whereas, the rates for 1–450 and 1–624 species could be explained by single exponentials, rates for the 1–908 and full-length species required two exponentials to adequately model their reaction kinetics (Supplementary Fig. S2; Table 1). Although the thermal reversion rate constants of full-length PhyB and the 1–908 fragment were comparable in magnitude (Table 1), the fast phase of the 1–908 species accounted for 90% of the reaction amplitude compared to 30% for full-length PhyB, leading to more rapid depletion of Pfr by this species (Fig. 2e). While this effect suggests that dimerization of full-length PhyB has a stabilizing effect on the Pfr state, increasing the concentration of the 1–908 fragment to favor dimerization had little to no effect on thermal reversion (Supplementary Fig. S3). This indicates that the HKRD stabilizes the slow kinetic phase, presumably by dampening thermal reversion of the Pfr-Pfr homodimer to the Pfr-Pr heterodimer^44^. The importance of the OPM in regulating the photostate of PhyB was further underscored as its removal to form the 1–624 fragment led to a 40-fold increase in the stability of Pfr (Fig. 2e; Table 1). Collectively, it appears that the PAS-PAS region destabilizes Pfr once formed, while the HKRD constrains Pr → Pfr photoconversion, and engenders a cooperative mechanism that stabilizes Pfr.

### Highly conserved residues in the NTE influence Pfr stability

**Figure 3:**
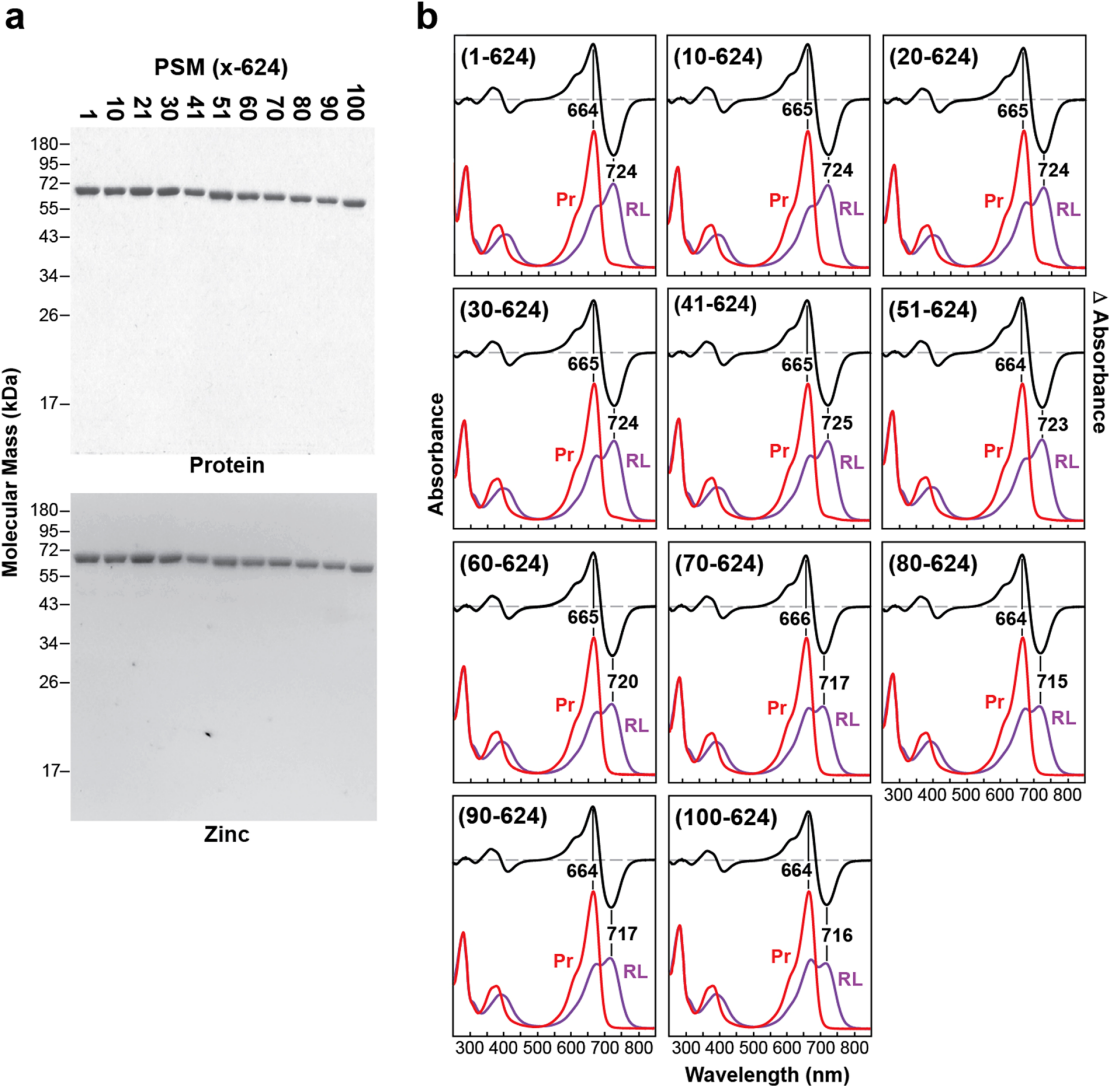
Spectroscopic properties of PSM mutants impacting the N-terminal extension (NTE) in PhyB. (**a**) Assembly of NTE truncations with PΦB. Purified PSM fragments were subjected to SDS-PAGE and either stained for protein with Coomassie Blue or for the bound bilin by zinc-induced fluorescence. For full-length images of the SDS-PAGE gels see Supplementary Fig. S1. **(b)** UV-Vis absorption and difference spectra of dark-adapted samples (Pr, red) and samples irradiated to steady state with 230 μmol⋅m^−2^⋅s^−1^ of 630-nm red light (RL, purple). The difference spectra are shown at 70% total amplitude. The absorption and difference maxima/minima are indicated.

**Figure 4:**
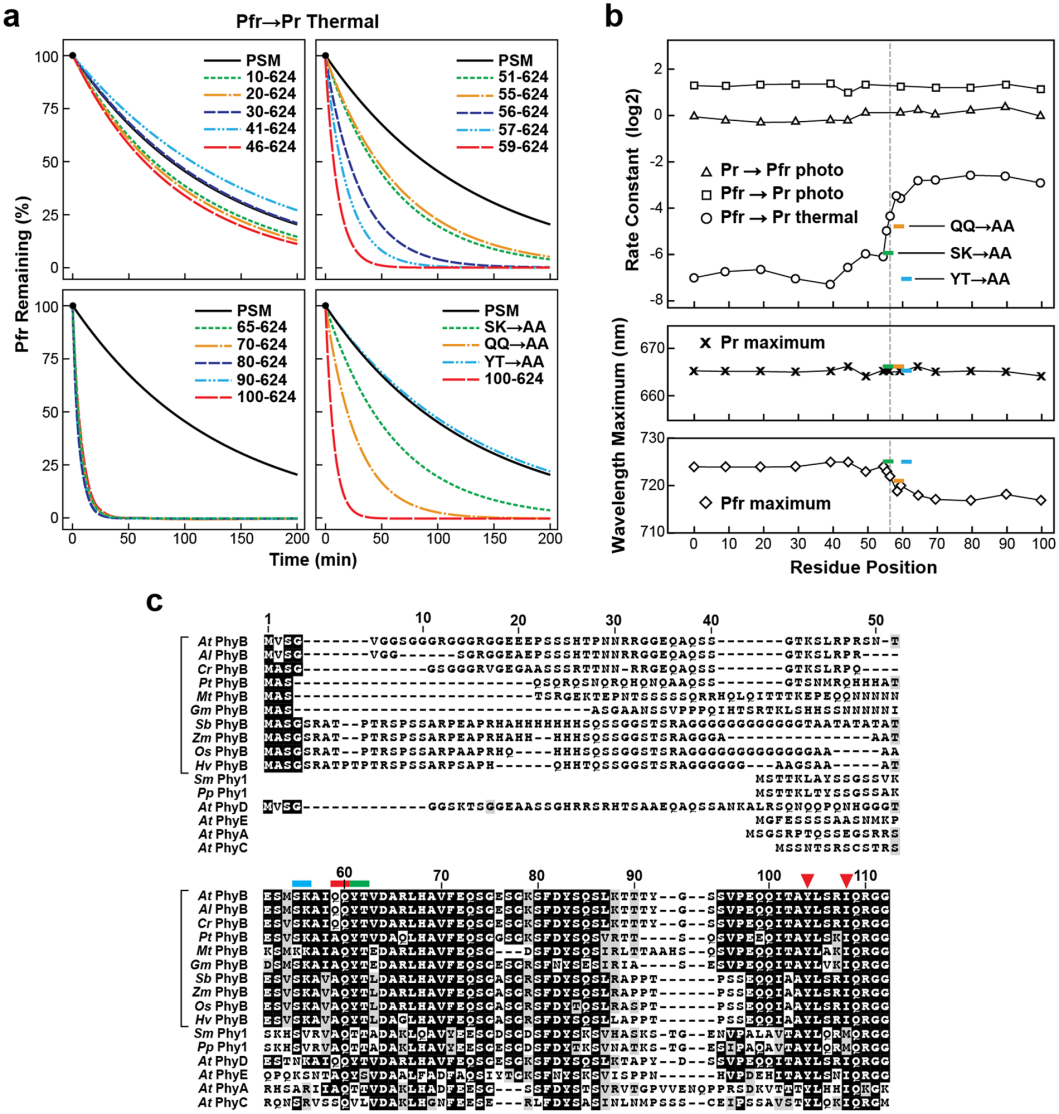
Region within the N-terminal extension (NTE) of PhyB influences the thermal reversion rate and absorption maximum of Pfr. Selected PSM fragments assembled with PΦB are described in Fig. 3. (**a**) Pfr → Pr thermal reversion kinetics of both N-terminal truncations and site-directed mutants modifying the NTE. The SK → AA, QQ → AA and YT → AA alanine substitutions impacted residues Ser55-Lys56, Gln59-Gln60, and Tyr61-Thr62, respectively (see panel (c)). Kinetic traces were calculated from rate constants listed in Table 1. (**b**) Spectral properties of the PSM mutants described in panel (a). Rates of Pr → Pfr and Pfr → Pr photoconversion and thermal reversion (top). Absorption maxima of Pr (middle) and Pfr (bottom). **(c)** Alignment of representation PhyB sequences (brackets) within the plant kingdom and with the other four Phy isoforms (PhyA, C, D and E) in Arabidopsis. Identical and similar amino acids are shown in black and grey boxes, respectively. Dashes denote gaps. Amino acid residue numbers for Arabidopsis PhyB are shown above. Positions of the substitution mutations are shown by colored bars. Red arrows highlight residues in the crystallographic structure of the 90–624 fragment of Arabidopsis PhyB (PDB: 4OUR^21^) that contact the bilin. Abbreviations: *At*, *Arabidopsis thaliana*; *Al*, *Arabidopsis lyrata*; *Cr*, *Capsella rubella*; *Pt*, *Populus trichocarpa*; *Mt*, *Medicago truncatula*; *Gm*, *Glycine max*; *Sb*, *Sorghum bicolor*; *Zm*, *Zea mays*; *Os*, *Oryza sativa*; *Hv*, *Hordeum vulgare*; *Sm*, *Selaginella moellendorffii; and Pp*, *Physcomitrella patens*.

Previous work with PhyA and PhyB implicated the NTE as important for protecting against thermal reversion and thus enhancing light detection^21,26–29,31,32^. To identify NTE residues that are critical, we first examined a series of mutants that sequentially delete ~10-amino-acid segments upstream of the knot and PAS domain. Importantly, all of the truncations expressed well, efficiently assembled with PΦB, and displayed normal Pr absorption, indicating that the NTE is not essential for acquisition of the Pr state (Fig. 3). However, whereas sequential trimming of the first 50 residues of the NTE had little impact on Pfr → Pr thermal reversion, subsequent deletions substantially accelerated the reaction by ~17 fold with a consistently fast rate seen for deletions missing residues 1–59 and beyond (Fig. 4a,b). Fine mapping located a critical region centered on residue 57, which was pin-pointed further to Gln59-Gln60 by alanine-scanning mutagenesis (Fig. 4a,b). The region also affected the absorption maximum of Pfr (without impact on that for Pr), which hypsochromically shifted from 724 nm to 717 nm as the region around residue 57 was eliminated or substituted (Fig. 4b). In contrast, comparable kinetics for Pr → Pfr and Pfr → Pr photoconversion were seen for these NTE mutants, indicating this region (or even the entire NTE) has little impact on bilin photochemistry (Table 1, Fig. 4b).

Interestingly, when we generated an alignment of Phy sequences from a variety of PhyB isoforms from seed and seedless plants along with the other four Phy isoforms from Arabidopsis, we discovered that the critical residues are within a patch best conserved within the PhyB subfamily, which also includes its *Brassicaceae* paralog PhyD (Fig. 4c)^36^. Notably, the NTE appears poorly conserved amongst all Phys just upstream of this region but becomes strongly conserved thereafter (i.e., residue 51 in Arabidopsis PhyB), suggesting that NTE features emerge after this dividing line that are important to all Phy lineages. Taken together, it appears that the NTE in PhyB is positioned to stabilize Pfr, and thus might have evolved in plants to counteract the destabilizing influence of the PAS-PAS and HKRD regions, or vice versa.

### PCB alters both spectroscopic properties and Pfr stability of PhyB

**Figure 5:**
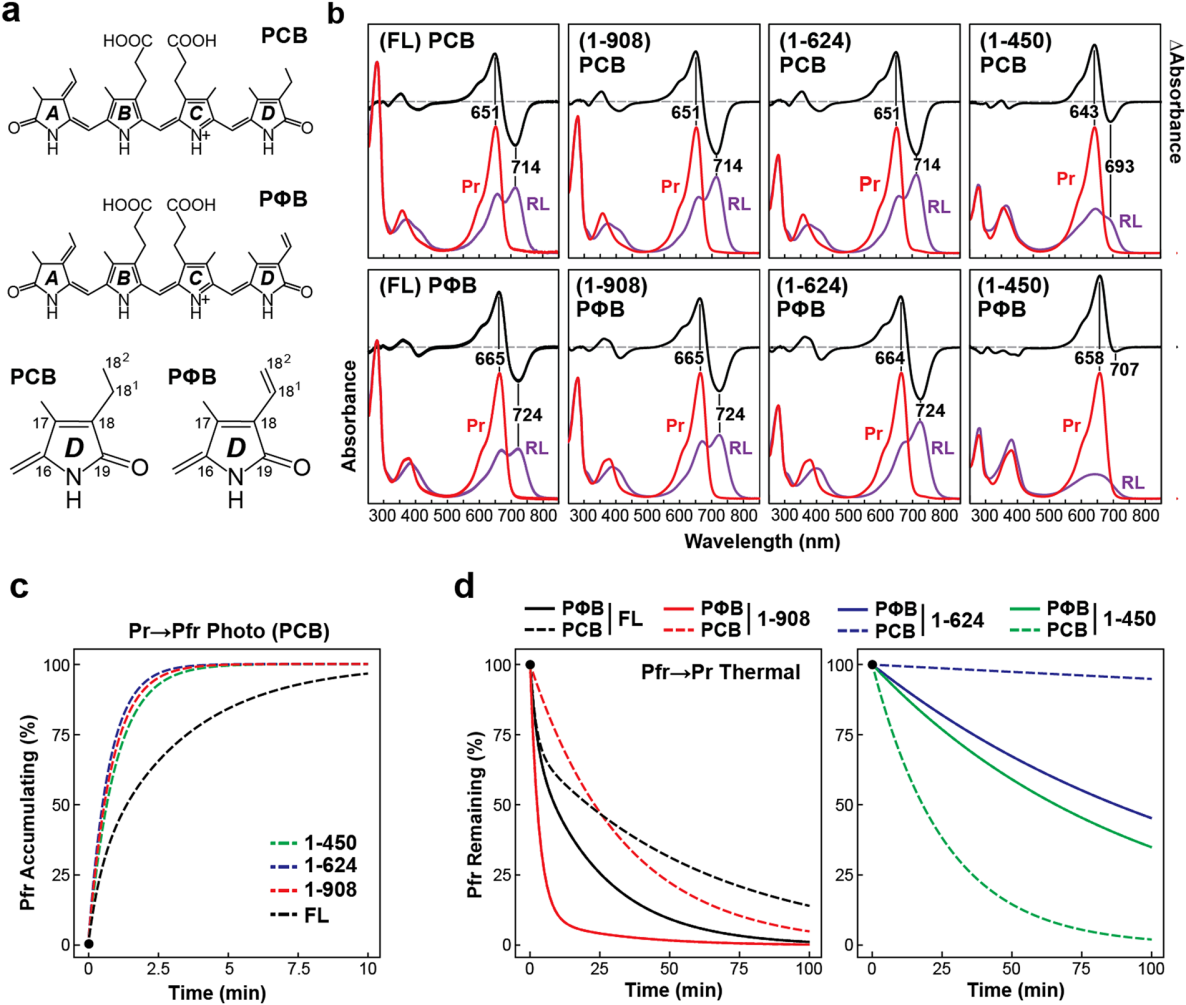
Substitution of PΦB with PCB dramatically alters the spectral properties of PhyB and thermal reversion of Pfr. **(a)** Chemical diagrams of PΦB and PCB. Differences in the D pyrrole rings are highlighted below. **(b)** UV-Vis absorption and difference spectra for full-length (FL) PhyB and various C-terminal truncation mutants after dark-adaption (Pr) or following saturating red-light irradiation (RL). To attain saturation of the Pfr state, the FL PΦB-containing species was irradiated with 660-nm light at 1250 μmol⋅m^−2^⋅s^−1^, whereas all other species were irradiated with 630-nm light at 230 μmol⋅m^−2^⋅s^−1^. Composition of the C-terminal truncations assembled with PΦB are described in Fig. 1a. Absorption maxima and minima are indicated. Bilin occupancies of the PCB- and PΦB-containing preparations are shown in Supplementary Fig. S4. **(c)** Pr → Pfr photoconversion of PCB-bound chromoproteins. **(d)** Thermal reversion rates for samples described in panel (b) upon assembly with PCB (dashed lines) or PΦB (solid lines). Kinetic traces were calculated from rate constants listed in Table 1.

Both its low cost and ready availability have made PCB an attractive alternative to PΦB in photochemical studies of Phys^22,41,45–49^,chemical complementation of PΦB synthesis mutants (e.g., Arabidopsis *hy2* alleles^50,51^), and the expanding collection of optogenetic strategies based on PhyB^34,35,38^. However, comparison of our data generated using PΦB relative to previous studies using PCB showed non-trivial photochemical differences in the behavior of PhyB. As judged by zinc-induced fluorescence of the bound bilins following SDS-PAGE, recombinant PhyB assembled with PCB with efficiencies equal to that seen for PΦB (Supplementary Fig. S4). Consistent with prior observations^41,52^, the Pr absorption spectra of full-length PhyB and the 1–908, and 1–624 fragments assembled with PCB all showed a 13- to 15-nm hypsochromic shift of the Q-band absorption maximum and an altered Soret band shape (Fig. 5b), which was caused, in part, by replacement of the C18 vinyl in PΦB with an ethyl moiety in PCB, which shortens the π-bond conjugation system of the bilin (Fig. 5a). The Q-band absorption maximum as Pfr was also shifted 10 nm.

Similar shifts were also seen for the 1–450 fragment as Pr, but surprisingly, while the 1–450 fragment assembled with PΦB generated only a bleached species following red light excitation, the fragment assembled with PCB generated as least some of a ‘Pfr’-like state whose Q-band absorption maximum (as determined from red/far-red light difference spectra) was hypsochromically shifted ~14 nm (693 nm) compared to the PΦB- containing form. Despite these shifts, the Pr → Pfr photoconversion rates for the PCB-containing preparations in red light were similar to those containing PΦB, including the substantially slower rate for the full-length chromoprotein (Fig. 5c).

More striking were the substantial changes in Pfr → Pr thermal reversion for PhyB assembled with PCB. For the full-length chromoproteins, the Pfr reversion rate was substantially slower for PCB- versus PΦB-containing preparations, where the slow rate constant was impacted to a greater extent than the faster rate constant (Fig. 5d; Table 1). Single exponential kinetics were seen for the 1–908 and 1–624 fragments, with the kinetic analyses indicating that the reversion rates were 11 and 15 fold slower, respectively, when PCB was incorporated versus PΦB. In contrast, thermal reversion for the 1–450 fragment flipped from those seen with the other constructions; instead of the PCB-containing species reverting slower than the PΦB-containing species, it reverted nearly 4 times faster (Fig. 5d; Table 1). Presumably, this fast reverting state reflects the Pfr-like species generated when the 1–450 fragment assembles with PCB (Fig. 5b). Taken together, the absence of the double bond at the C18 side chain in PCB versus PΦB does more to influence the kinetic parameters of PhyB than simply blue-shifting absorption of its Pr and Pfr states.

## Discussion

Phys are unique among photoreceptors by their ability to assess numerous environmental parameters to generate a coherent response based on the amount of Pfr generated, rates of Pr/Pfr interconversion, and the final acquired ratio of the two states. These factors are interconnected through the relative magnitudes of the rate constants for photoconversion and thermal reversion, the temperature dependence of each (predominantly thermal reversion), and the intensity and color of the impinging light. Through these parameters, Phys can measure: (i) fluence rate (intensity), (ii) fluence (photon counting), (iii) fluence duration (time), (iv) shading by detecting alterations in the Pr/Pfr ratio caused by changes in the ratio of impinging red and far-red light, (v) temperature through enthalpic effects of Pfr → Pr reversion, and possibly (vi) the annual changes in photoperiod by diurnally quantifying the nighttime loss of Pfr (hourglass)^3–6^. How Phys respond to all these features is not completely understood. Certainly, one strategy by plants  is to link individual Phys with specific sensory transduction chains to engender distinct signaling outcomes. Another would be to adjust the Pr/Pfr transitions of Phys mechanically to imbue unique signaling potentials. For the latter strategy, Phy interconversion could be viewed as a delicate balance among several features both proximal and substantially distal to the chromophore that modify exchange between the two endstates. By adjusting these features even for Phy isoforms within the same organism, a rich panoply of signaling potentials could be possible, thus enabling broad environmental perception, including measurement of light, temperature, and possibly time.

Numerous studies have revealed a critical role for the bilin/protein contacts within the chromophore-binding pocket of the GAF domain in generating the unusual absorption properties of Phys as compared to those of free bilins, and for impacting photoconversion and thermal reversion^14,15,21–24^. Here, we extend this influence to the NTE, which had been previously documented to accelerate thermal reversion when removed^21,29^. Fine mapping of the NTE pin-pointed the causative motif to a region centered on Gln59-Gln60. This motif not only strongly impacts thermal reversion but also bathochromically shifts the absorption of Pfr, implying an influence on the bilin/protein interface.

How the NTE induces these effects on a chromophore almost completely enveloped within the GAF domain remains unclear in the absence of a 3D structure. Structural details for the NTE immediately upstream of the PAS domain (residues 90–100) suggest that it folds back on the PAS/GAF domains^21^, which might allow residues 50–60 to interact with amino acids in intimate contact with the bilin or with the PHY-domain hairpin in close proximity. In support, recent hydrogen/deuterium exchange mass spectrometry revealed that the conformation of the NTE and/or its association with the GAF domain are altered upon photoconversion^46^. Notably, Pfr-enhanced deuterium uptake was seen for residues 55–58 and 62–87, which is indicative of increased solvent accessibility. One likely scenario is that Pfr formation releases these NTE residues from a buried state to form new more solvent-exposed contacts that enhance Pfr stability. As implied by our truncation studies, absence of these contacts then destabilizes Pfr. Intriguingly, the NTE of PhyB (and possibly other plant Phys) is modified by phosphorylation. In particular, modifications at Ser86 and Tyr104 not only impacts signaling by the photoreceptor but also substantially accelerates thermal reversion^28,32^, thus implicating this NTE/GAF domain contact as an important control point for regulating Pfr signal strength.

Even more dramatic are the allosteric effects of the C-terminal domains on PhyB photochemistry and thermal reversion despite their substantial distance away from the bilin-binding pocket (Fig. 1e)^15,33^. Whereas removal of the HKRD dramatically accelerates photoconversion, subsequent deletions of the PAS-PAS region and HKRD regions combine to dramatically dampen thermal reversion, implying that the OPM constrains photoconversion to Pfr and then destabilizes Pfr once formed. Presumably, the sum total of these changes would substantially desensitize PhyB to light. The influences of the OPM on these parameters could reflect structural features within these domains that directly impinge on the PSM. Possibilities include altering the helical spine that transverses nearly the entire length of the polypeptide and/or adjusting the splay/twist reorientation of the OPM relative to the PSM as the paired PHY domains attempt to separate due to the photoconversion-induced hairpin contraction^1^. Alternatively, the effects could reflect the importance of dimerization to Pfr stability, as has been implicated from similar truncation studies of *D*. *radiodurans* BphP with its canonical HK OPM^19^. In support of the latter, Klose *et al*.^44^ recently provided evidence that dimerization through the HKRD leads to cooperativity in thermal reversion; *i*.*e*., when one subunit as Pfr in the Phy dimer reverts to Pr, the reversion rate of the second becomes faster.

Our *in vitro* studies detected a much more rapid rate of thermal reversion for full-length PhyB than that measured *in planta* ^44^, suggesting that the cellular environment also impacts the process. Included are the aforementioned modification of the NTE by phosphorylation, and/or the interaction of PhyB with other signaling components, including PIFs that were recently shown to strongly influence thermal reversion *in vivo* ^53^. Conceivably, other modifications and binding partners could alter photoconversion and/or thermal reversion rates leading to a highly regulated light-feedback system that can dampen or enhance the photosensitivity of PhyB in response to external conditions.

At the most proximal level is the nature of the bilin and how it fits within the GAF domain pocket. Strikingly, the Pfr state is substantially destabilized when PhyB is assembled with its natural bilin PΦB (with its more rigid C18 vinyl moiety) as compared to that assembled with PCB used by cyanobacterial Phys (with its more flexible C18 ethyl moiety (see Fig. 5a)). Notably, this bilin-dependent difference was also observed by Remberg *et al*.^54^ studying the PSM of potato PhyB expressed recombinantly in yeast, implying that the effects could be universal among plant Phys. Modeling PCB into the 3D structure of the PhyB PSM^21^ suggests that one consequence is the ethyl group having more conformational freedom to interact with neighboring amino acids. As Pr, the C18 vinyl group of PΦB is tightly flanked by Met274, Tyr276, Val286, Tyr303, and Met365. Fitting PCB into this position predicted that its ethyl group unlikely perturbs this solvent-excluded hydrophobic interaction (Supplementary Fig. S5). However, when the bilin cavity of *D*. *radiodurans* BphP as Pfr was used as the template to model the bilin-binding pocket of PhyB^14^, a substantially strained D ring was seen after the 15*Z* to 15*E* isomerization. Here, the vinyl moiety of PΦB was now flanked by Leu301, Tyr361 and Met365 of the GAF domain, and Pro581 of the hairpin, while a small hydrophobic cavity appeared near Met274, Tyr276, Val286, Tyr298, and Tyr303 (Supplementary Fig. S5). Presumably, the extra degrees of rotational freedom bestowed by the ethyl group in PCB would allow the D-ring as Pfr to nestle into this hydrophobic pocket as a lower energy state, thus providing increased resilience against isomerization back to 15*Z* for the Pr state. Similar effects have also been seen when saturated carbon functional groups of various lengths were used to replace the C18 vinyl moiety^55^, suggesting that rotational freedom in combination with overall hydrophobicity of this feature enhances Pfr stability.

It is commonly appreciated that plant Phys evolved from using PCB, as in cyanobacterial Phys, to PΦB to better align their absorption with those of chlorophylls and thus enhance detection of shade. A second possibility emerging from our data is that the switch to PΦB also enhanced the use of Pfr → Pr thermal reversion as an environmental signal. Whereas the reversion rate of Arabidopsis PhyB assembled with PΦB appears sufficiently fast relative to the Pr → Pfr photoconversion rate to enable thermoperception and the diurnal measurement of darkness, the reversion rate for PhyB assembled with PCB might be too slow to be physiologically relevant. For example, point mutants of Arabidopsis PhyB that significantly dampen thermal reversion lack temperature-dependent responses at physiological temperatures^3^.

Coincidently, PCB is commonly used as the chromophore for optogenetic applications involving PhyB^34,35,56,57^. While this choice has benefited strategies where a stable Pfr species is needed, the alternative use of PΦB might avail new applications where a decelerating Pfr signal would help. Our finding that the 1–908 truncation of PhyB has an innate tendency to form dimers should also be considered when using this truncation in optogenetic applications that require a monomeric photoreceptor. Thus, the expression of the 1–908 fragment might need to be controlled to avoid dimerization-induced artifacts.

The accumulating structural data for Phys indicate that they are highly modular dimeric receptors with numerous features allosterically contributing, either positively or negatively, to their signaling potential even at a distance. Presumably by adjusting the balance between these features, Phys could evolve novel isoforms capable of detecting specific environmental signals. For plant Phys, one can imagine that thermal reversion was selectively accelerated in the PhyB isoform to effectively compete with the rates of photoconversion, thus enabling better measurement of photoperiod and temperature, and signaling in the light-rich environments of mature plants, while PhyA could have dampened thermal reversion to better detect the low light levels seen by germinating seedlings. One can even speculate that adjusting the interconversion balance between Pr and Pfr led to the extreme properties found for bacterial bathy-Phys that generate a dark-adapted Pfr state without photoexcitation^1,17,58^. Clearly, further spectroscopic characterizations of Phys in the contexts of their photo- and thermosensing and downstream signaling should reveal this evolutionary interplay, which in turn can be exploited to engineer Phys with novel environmental signaling potentials.

## Methods

### Recombinant expression of Arabidopsis PhyB

An intron-less *A*. *thaliana PhyB* gene designed for recombinant expression was appended to codons for an N-terminal 6His tag followed by a TEV protease cleavage site (MGSSHHHHHHSSENLYFQG), and inserted into the modified pBAD Myc-His plasmid (Invitrogen)^21^. Truncations were generated by the PIPES^59^ or Gibson assembly methods^60^, whereas the site-directed mutants were generated by Quickchange mutagenesis (Stratagene) in combination with appropriate oligonucleotide primers. The 6His-TEV-PhyB apoproteins were co-expressed in *E*. *coli* BL21(DE3) cells with the pL-PΦB or pL-PCB plasmids harboring genes encoding the *Synechocystis* PCC6803 heme oxygenase (Ho1), and either the Arabidopsis HY2 or *Synechocystis* PCC6803 PcyA synthases that direct production of PΦB or PCB, respectively^21,48,61^. Cells were first grown at 37 °C to 1 OD_600nm_ in Terrific Broth containing 1 mM MgSO_4_ and 250 μM 5-aminolevulinic acid, and incubated for an additional hour at 16 °C. Isopropyl β-D-1-thiogalactopyranoside was then added to 1 mM to induce the expression of the PΦB or PCB biosynthetic genes, which was followed one hour later by addition of 0.2% L-arabinose to induce expression the PhyB polypeptide^21^. Cultures were grown overnight at 16 °C, harvested by centrifugation, and immediately frozen at liquid nitrogen temperatures. Unless otherwise noted, all culture growths, purification of PhyB as Pr, and subsequent manipulations of samples were performed at 0–4 °C in darkness or under dim green safelight conditions.

### Purification of PhyB

PhyB-containing cell pellets were thawed and disrupted by sonication in a fourfold excess of lysis buffer (LB: 10% glycerol, 20 mM HEPES-NaOH (pH 7.8), 500 mM NaCl, 1 mM 2-mercaptoethanol, 0.05% Tween 20, 1 mM phenylmethanesulfonyl fluoride, one tablet per liter Roche or Pierce complete EDTA-free protease inhibitor) also containing 30 mM imidazole. Cell lysates were clarified by centrifugation at 35,000 x*g*, and followed by filtration through a G-25 Sephadex column equilbrated in LB. The filtrate was loaded onto a Ni^2+^-nitrilotriacetic acid (Ni-NTA) column (GE) pre-equilibrated in LB containing 30 mM imidazole, washed with the same buffer, and the bound proteins were eluted with LB containing 300 mM imidazole. Fractions containing PhyB were exchanged by ultrafiltration (Amicon Ultra-15 filters) into anion exchange buffer (AEB: 10% glycerol, 20 mM HEPES-NaOH (pH 7.8), and 10 mM 2-mercaptoethanol) also containing 20 mM NaCl, and subjected to anion exchange chromatography using a HiTrap Q Sepharose column (GE) in combination with a 20–500 mM linear gradient of NaCl in AEB. PhyB-containing fractions were pooled, exchanged into LB, and subjected to a second round of Ni-NTA chromatography using a 30–140 mM linear gradient of imidazole in LB.

Purified PhyB fractions as Pr were incubated for three hours at 4 °C with ~1 mg of 6His-TEV protease. Subtractive nickel affinity chromatography was conducted by exchanging samples back into LB containing 30 mM imidazole and passing them through a Ni-NTA column equilibrated in LB to retain the 6His-TEV protease. PhyB samples were concentrated and flash frozen to liquid nitrogen temperatures as 30 µL drops. Prior to analysis, thawed samples were passed through a Superdex 200 column to provide exchange into the appropriate buffers and to remove contaminants and PhyB breakdown products. Bilin occupancy was assessed by zinc-induced-fluorescence of the chromoproteins following SDS-PAGE^9^.

TEV protease bearing a 6His tag was expressed in BL21(DE3) cells, and enriched via Ni-NTA chromatography as above, exchanged into cation exchange buffer (CEB: 0.3 mM tris(2-carboxyethyl)phosphine), and 10 mM Tris-HCl (pH 7.5)), and subjected to HiTrap SP Sepharose (GE) chromatography (GE) using a 0–600 mM linear gradient of NaCl in CEB^62^. Glycerol was added to the TEV protease preparations (~1 mg/ml) at a 50% (w/v) concentration, and stored at −20 °C.

### Size exclusion chromatography (SEC)

SEC (0.3 ml/min flow rate) of full-length PhyB and various truncations was conducted at 10 °C using an ÄKTA FPLC (GE Healthcare) equipped with the Superdex 200 HR 10/30 column (GE) equilibrated in 150 mM KCl, 50 mM HEPES-NaOH (pH, 7.8), and 0.3 mM tris(2-carboxyethyl)phosphine. Prior to loading, samples (100 µL) were fully converted to Pr using far-red (730 nm) light. Apparent molecular mass based on elution volume was calculated using Unicorn software (GE Healtcare) in combination with size standards (BioRad).

### Single particle electron microscopy

2D class averages of PhyB as Pr were generated by negative staining electron microscopy. Full-length PhyB or the 1–908 truncation were fixed onto carbon films, stained with phosphotungstic acid, and imaged by a JEOL JEM–2010F transmission electron microscope (JEOL Ltd., Tokyo, Japan) operating at 200 kV as described previously^15,33^. Micrographs were recorded in a low dose mode (15 electrons/Å^2^) at a 50,000x magnification using a Gatan (Pleasanton, California) UltraScan 4000 CCD camera (4096 × 4096 pixels), which corresponded to 2.12-Å/pixel sampling at the specimen level. Particle selection and image processing were conducted using the EMAN and EMAN2 software packages as described previously^15^.

### UV-Vis spectroscopy and kinetic analyses

For all spectroscopic experiments, the PhyB samples were exchanged into 50 mM HEPES-NaOH (pH 7.9), 1 mM Na_4_EDTA, 10 mM 2-mercaptoethanol, and 150 mM KCl. Absorption spectra as Pr or following various fluences of red light (630 nm) were collected at 25 °C with a Cary 60 spectrophotometer (Agilent). Unless specified, Pfr spectra were recorded after steady state had been achieved using continuous 630 nm irradiations. Pr → Pfr and Pfr → Pr photoconversions were driven with LED lights having peak emissions of 660 nm or 740 nm, respectively, using a LiCor LI-185B photometer to determine fluence rate. For all photoconversion reactions the sample concentration was adjusted so the maximal absorption of the Q band was 0.4 as Pr. To ensure consistency between measurements the same cuvette was used for all experiments and was loaded with the same volume of sample. Pfr → Pr thermal reversion was measured at 25 °C in the dark. Time-resolved spectra were collected from 630–850 nm in 10-nm intervals, and the scan rate was set at 24,000 nm/min to minimize the red- or far-red light exposure. Examples of the raw kinetic traces can be found in Supplementary Fig. S2. Kinetic rate constants for interconversion were calculated in R using a global fit of the absorption data at 640, 650, 660, 670, 700, 710, 720, 730 and 740 nm together with the equation Abs_i = _ΔAbs_i_⋅exp(-k⋅t) + Abs(0)_i_, where i represents the ith wavelength, Abs is the absorption, ΔAbs is the reaction amplitude, k is the rate constant, t is time, and Abs(0) is the absorption at time zero. If two exponentials were required to explain the results, the data were fit to a simple sum of two exponentials using the equation described above.

### Phy sequence alignment

Amino-acid-sequence alignment of representative members of the plant Phy family was generated using the Clustal Omega server^63^ and displayed with BoxShade (http://www.ch.embnet.org/software/BOX_form.html). The extreme N-terminal sequences, which are poorly conserved, were manually compressed to ease viewing.

## Electronic supplementary material

Supplementary Information

## References

[1] Burgie ES, Vierstra RD (2014). Phytochromes: an atomic perspective on photoactivation and signaling. Plant Cell.

[2] Rockwell NC, Su YS, Lagarias JC (2006). Phytochrome structure and signaling mechanisms. Annu. Rev. Plant Biol..

[3] Legris M (2016). Phytochrome B integrates light and temperature signals in Arabidopsis. Science.

[4] Jung JH (2016). Phytochromes function as thermosensors in Arabidopsis. Science.

[5] Devlin, P. F. Circadian regulation of photomorphogenesis *In: Photomorphogenesis in Plants and Bacteria* E. Schafer and F. Nagy, eds Dordrecht, The Netherlands: Springer, pp. 567–604. (2006).

[6] Schäfer, E. & Nagy, F. *Photomorphogenesis in plants and bacteria*. Vol. 3rd Edn (Springer, 2006).

[7] Franklin KA, Quail PH (2010). Phytochrome functions in *Arabidopsis* development. J. Exp. Bot..

[8] Auldridge ME, Forest KT (2011). Bacterial phytochromes: more than meets the light. Crit. Rev. Biochem. Mol. Biol..

[9] Bhoo SH, Davis SJ, Walker J, Karniol B, Vierstra RD (2001). Bacteriophytochromes are photochromic histidine kinases using a biliverdin chromophore. Nature.

[10] Wagner JR, Brunzelle JS, Forest KT, Vierstra RD (2005). A light-sensing knot revealed by the structure of the chromophore-binding domain of phytochrome. Nature.

[11] Yeh KC, Wu SH, Murphy JT, Lagarias JC (1997). A cyanobacterial phytochrome two-component light sensory system. Science.

[12] Rockwell NC, Lagarias JC (2017). Phytochrome diversification in cyanobacteria and eukaryotic algae. Curr. Opin. Plant Biol..

[13] Takala H (2014). Signal amplification and transduction in phytochrome photosensors. Nature.

[14] Burgie ES, Zhang J, Vierstra RD (2016). Crystal structure of *Deinococcus* phytochrome in the photoactivated state reveals a cascade of structural rearrangements during photoconversion. Structure.

[15] Burgie ES (2014). Crystallographic and electron microscopic analyses of a bacterial phytochrome reveal local and global rearrangements during photoconversion. J. Biol. Chem..

[16] Anders K, Daminelli-Widany G, Mroginski M, von Stetten D, Essen L (2013). Structure of the cyanobacterial phytochrome 2 photosensor implies a tryptophan switch for phytochrome signaling. J. Biol. Chem..

[17] Yang X, Kuk J, Moffat K (2009). Conformational differences between the Pfr and Pr states of *Pseudomonas aeruginosa* bacteriophytochrome. Proc. Natl. Acad. Sci. USA.

[18] Cornilescu CC (2014). Dynamic structural changes underpin photoconversion of a blue/green cyanobacteriochrome between its dark and photoactivated states. J. Biol. Chem..

[19] Takala H, Bjorling A, Linna M, Westenhoff S, Ihalainen JA (2015). Light-induced changes in the dimerization interface of bacteriophytochromes. J. Biol. Chem..

[20] Bjorling A (2016). Structural photoactivation of a full-length bacterial phytochrome. Sci. Adv..

[21] Burgie ES, Bussell AN, Walker JM, Dubiel K, Vierstra RD (2014). Crystal structure of the photosensing module from a red/far-red light-absorbing plant phytochrome. Proc. Natl. Acad. Sci. USA.

[22] Oka Y, Matsushita T, Mochizuki N, Quail PH, Nagatani A (2008). Mutant screen distinguishes between residues necessary for light-signal perception and signal transfer by phytochrome B. PLoS Genet..

[23] Wagner JR (2008). Mutational analysis of *Deinococcus radiodurans* bacteriophytochrome reveals key amino acids necessary for the photochromicity and proton exchange cycle of phytochromes. J. Biol. Chem..

[24] Hahn J (2006). Probing protein-chromophore interactions in Cph1 phytochrome by mutagenesis. FEBS J..

[25] Takala H, Lehtivuori H, Hammaren H, Hytonen VP, Ihalainen JA (2014). Connection between absorption properties and conformational changes in *Deinococcus radiodurans* phytochrome. Biochemistry.

[26] Zhang J, Stankey RJ, Vierstra RD (2013). Structure-guided mutagenesis of plant phytochrome B with altered photochemistry and light signaling. Plant Physiol..

[27] Adam E (2011). Altered dark- and photoconversion of phytochrome B mediate extreme light sensitivity and loss of photoreversibility of the *phyB-401* mutant. PLoS One.

[28] Medzihradszky M (2013). Phosphorylation of phytochrome B inhibits light-induced signaling via accelerated dark reversion in *Arabidopsis*. Plant Cell.

[29] Cherry JR, Hondred D, Walker JM, Vierstra RD (1992). Phytochrome requires the 6-kDa N-terminal domain for full biological activity. Proc. Natl. Acad. Sci. USA.

[30] Sweere U (2001). Interaction of the response regulator ARR4 with phytochrome B in modulating red light signaling. Science.

[31] Hajdu A (2015). High-level expression and phosphorylation of phytochrome B modulates flowering time in Arabidopsis. Plant J..

[32] Nito K, Wong CC, Yates JR, Chory J (2013). Tyrosine phosphorylation regulates the activity of phytochrome photoreceptors. Cell. Rep..

[33] Li H, Zhang J, Vierstra RD (2010). Quaternary organization of a phytochrome dimer as revealed by cryoelectron microscopy. Proc. Natl. Acad. Sci. USA.

[34] Levskaya A, Weiner OD, Lim WA, Voigt CA (2009). Spatiotemporal control of cell signalling using a light-switchable protein interaction. Nature.

[35] Toettcher JE, Weiner OD, Lim WA (2013). Using optogenetics to interrogate the dynamic control of signal transmission by the Ras/Erk module. Cell.

[36] Mathews S (2010). Evolutionary studies illuminate the structural-functional model of plant phytochromes. Plant Cell.

[37] Ni W (2014). A mutually assured destruction mechanism attenuates light signaling in Arabidopsis. Science.

[38] Shimizu-Sato S, Huq E, Tepperman JM, Quail PH (2002). A light-switchable gene promoter system. Nat. Biotechnol..

[39] Cherry JR (1993). Carboxy-terminal deletion analysis of oat phytochrome A reveals the presence of separate domains required for structure and biological activity. Plant Cell.

[40] Lagarias JC, Mercurio FM (1985). Structure function studies on phytochrome. Identification of light-induced conformational changes in 124-kDa *Avena* phytochrome *in vitro*. J. Biol. Chem..

[41] Elich TD, Chory J (1997). Biochemical characterization of Arabidopsis wild-type and mutant phytochrome B holoproteins. Plant Cell.

[42] Karniol B, Wagner JR, Walker JM, Vierstra RD (2005). Phylogenetic analysis of the phytochrome superfamily reveals distinct microbial subfamilies of photoreceptors. Biochem J..

[43] von Stetten D (2007). Highly conserved residues D197 and H250 in Agp1 phytochrome control the proton affinity of the chromophore and Pfr formation. J. Biol. Chem..

[44] Klose C (2015). Systematic analysis of how phytochrome B dimerization determines its specificity. Nat. Plants.

[45] Kikis EA, Oka Y, Hudson ME, Nagatani A, Quail PH (2009). Residues clustered in the light-sensing knot of phytochrome B are necessary for conformer-specific binding to signaling partner PIF3. PLoS Genet..

[46] von Horsten S (2016). Mapping light-driven conformational changes within the photosensory module of plant phytochrome B. Sci. Rep..

[47] Kunkel T, Speth V, Buche C, Schafer E (1995). *In vivo* characterization of phytochrome-phycocyanobilin adducts in yeast. J. Biol. Chem..

[48] Gambetta G, Lagarias J (2001). Genetic engineering of phytochrome biosynthesis in bacteria. Proc. Natl. Acad. Sci. USA.

[49] Yeh K, Lagarias J (1998). Eukaryotic phytochromes: light-regulated serine/threonine protein kinases with histidine kinase ancestry. Proc. Natl. Acad. Sci. USA.

[50] Kami C (2004). Complementation of phytochrome chromophore-deficient Arabidopsis by expression of phycocyanobilin:ferredoxin oxidoreductase. Proc. Natl. Acad. Sci. USA.

[51] Parks BM, Quail PH (1991). Phytochrome-deficient*hy1* and *hy*2 long hypocotyl mutants of Arabidopsis are defective in phytochrome chromophore biosynthesis. Plant Cell.

[52] Wahleithner JA, Li LM, Lagarias JC (1991). Expression and assembly of spectrally active recombinant holophytochrome. Proc Natl. Acad. Sci. USA.

[53] Smith, R. W. *et al*. Interactions between phyB and PIF proteins alter thermal reversion reactions *in vitro*. *Photochem*. *Photobiol*., 10.1111/php.12793 (2017). 10.1111/php.12793 28503745

[54] Remberg A, Ruddat A, Braslavsky SE, Gartner W, Schaffner K (1998). Chromophore incorporation, Pr to Pfr kinetics, and Pfr thermal reversion of recombinant N-terminal fragments of phytochrome A and B chromoproteins. Biochemistry.

[55] Robben U, Lindner I, Gartner W (2008). New open-chain tetrapyrroles as chromophores in the plant photoreceptor phytochrome. J. Am. Chem. Soc..

[56] Chernov KG, Redchuk TA, Omelina ES, Verkhusha VV (2017). Near-infrared fluorescent proteins, biosensors, and optogenetic tools engineered from phytochromes. Chem. Rev..

[57] Adrian M, Nijenhuis W, Hoogstraaten RI, Willems J, Kapitein LC (2017). A phytochrome-derived photoswitch for intracellular transport. ACS Synth. Biol..

[58] Karniol B, Vierstra RD (2003). The pair of bacteriophytochromes from *Agrobacterium tumefaciens* are histidine kinases with opposing photobiological properties. Proc. Natl. Acad. Sci. USA.

[59] Klock HE, Koesema EJ, Knuth MW, Lesley SA (2007). Combining the polymerase incomplete primer extension method for cloning and mutagenesis with microscreening to accelerate structural genomics efforts. Proteins: Struct. Funct. Bioinf..

[60] Gibson DG (2009). Enzymatic assembly of DNA molecules up to several hundred kilobases. Nat. Methods.

[61] Fischer AJ (2005). Multiple roles of a conserved GAF domain tyrosine residue in cyanobacterial and plant phytochromes. Biochemistry.

[62] Blommel PG, Fox BG (2007). A combined approach to improving large-scale production of tobacco etch virus protease. Protein Expr. Purif..

[63] McWilliam H (2013). Analysis tool web services from the EMBL-EBI. Nucleic Acids Res..

