# Supplementary material for: Photosensing and Thermosensing by Phytochrome B Require Both Proximal and Distal Allosteric Features within the Dimeric Photoreceptor
Source: Sci Rep. 2017 Oct 20;7:13648. doi: 10.1038/s41598-017-14037-0 (PMC5651913; doi:10.1038/s41598-017-14037-0)
Supplement: Supplementary file 1 — Supplementary Information [file 41598_2017_14037_MOESM1_ESM.pdf]

SUPPLEMENTARY INFORMATION

Photosensing and Thermosensing by Phytochrome B Require Both Proximal and Distal Allosteric Features within the Dimeric Photoreceptor

E. Sethe Burgie, Adam N. Bussell, Shu-Hui Lye, Tong Wang, Weiming Hu, Katrice E. McLoughlin, Erin L. Weber, Huilin Li, and Richard D. Vierstra

Figure 1b.

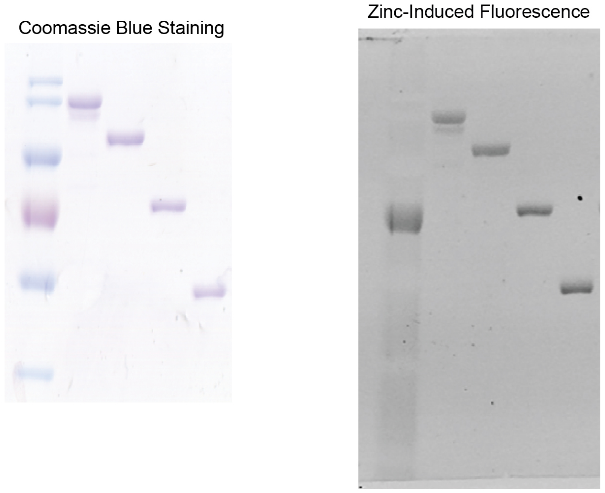

Figure 3a.

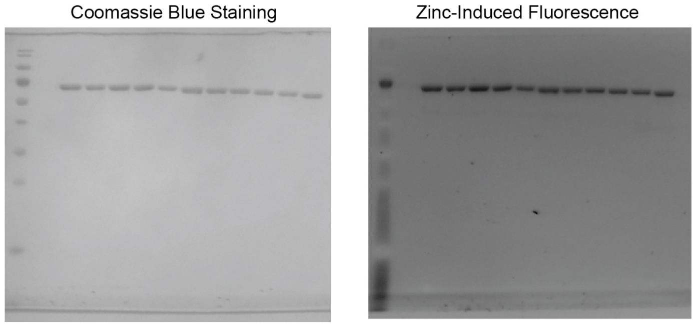

Supplemental Figure 4.

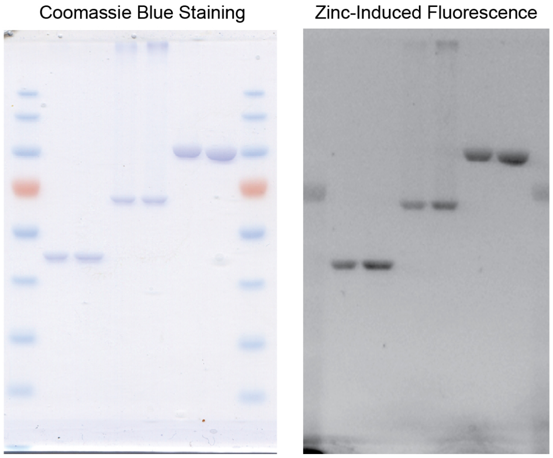

Supplementary Figure S1. Full-length images of the SDS-PAGE gels shown in Figures 1b and 3a, and Supplemental Figure S4. The gels in each figure were truncated for simplicity. Here, the full length of the gels are shown.

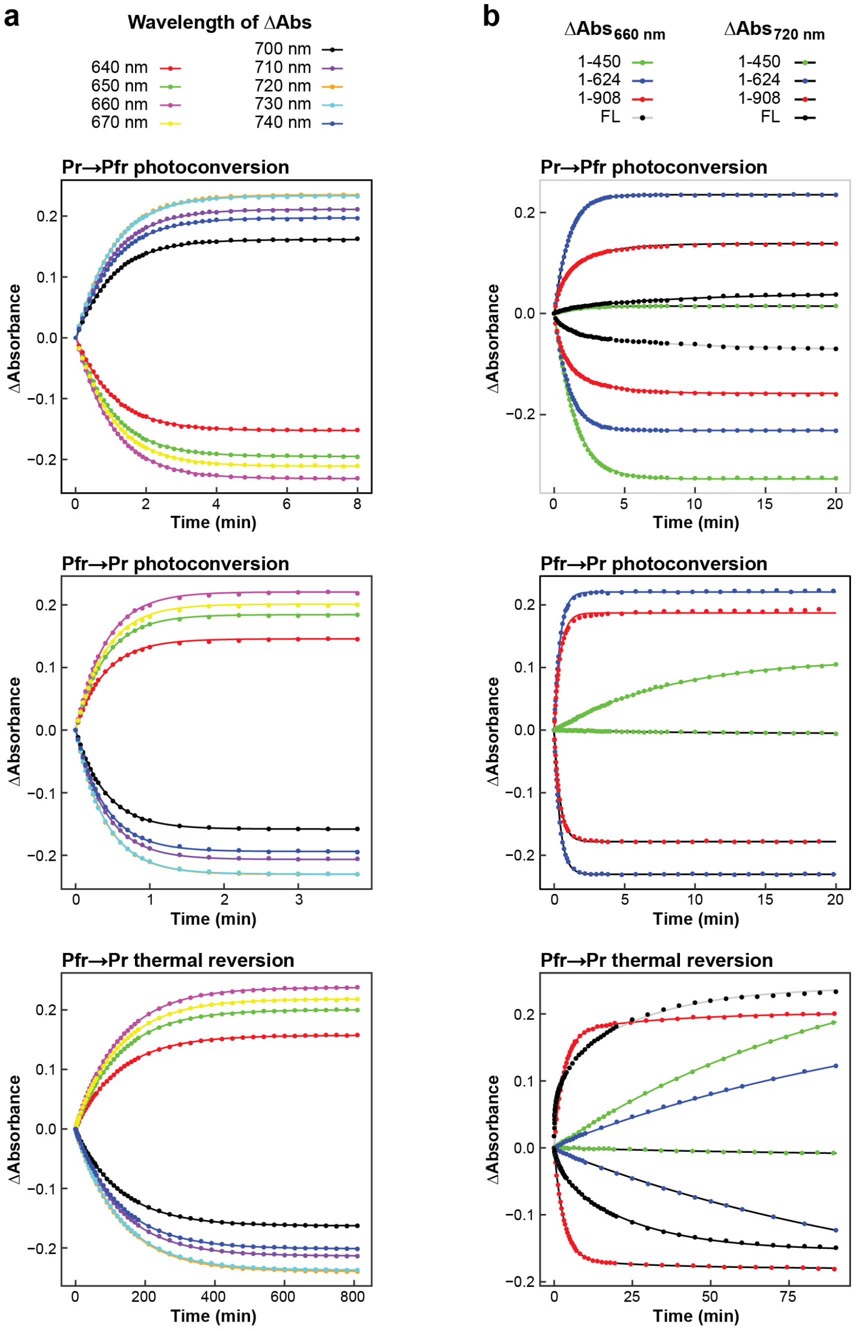

Supplementary Figure S2. Global fitting of multiple wavelengths was employed to obtain robust rate constants for the Arabidopsis PhyB truncation mutants. (a) Representative fitting for Pr→Pfr and Pfr→Pr photoconversion, and Pfr→Pr thermal reversion of the 1-624 fragment of PhyB. Datapoints show the measured change in absorbance at a given wavelength as a function of time, and the lines show the global fits to the data. **(b)** Rate constants for all Phy truncations in this study were measured as per panel (a). Representative datapoints and their simulation from the global fits at 660 nm and 720 nm are shown.

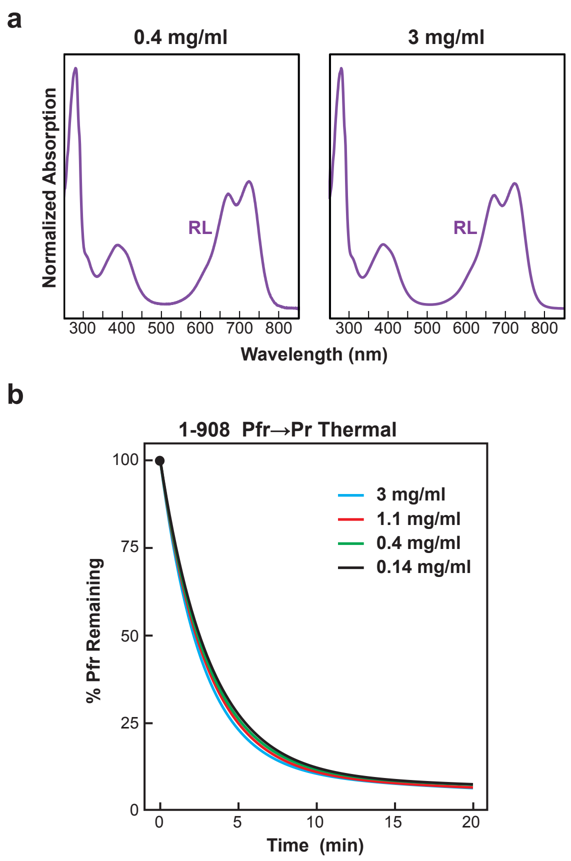

Supplementary Figure S3. Concentration dependence of photoconversion and thermal reversion of the 1-908 fragment of Arabidopsis PhyB. (a) Photoconversion from Pr to Pfr by 230 $\mu mol \cdot m^{-2} \cdot s^{-1}$ of 630-nm red light (RL) using low and high concentrations of the 1-908 fragment. Spectra were normalized by absorption at 280 nm. **(b)** Thermal reversion at 25°C of Pfr back to Pr for the 1-908 fragment at various concentrations. Simulations of the reaction determined from the global fits to the data are shown.

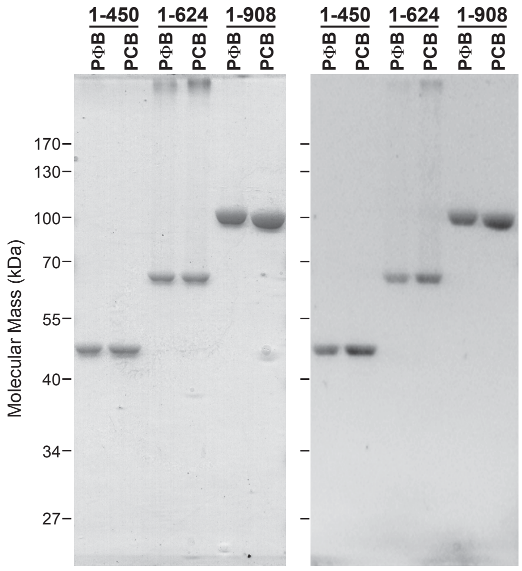

Supplementary Figure S4. PCB and PΦB are incorporated with equivalent efficiency in various PhyB truncations. Recombinantly PhyB samples were assembled with the bilins in *E. coli* and purified. The preparations were then subjected to SDS-PAGE and either stained for protein by Coomassie staining (left panel), or assayed for the bound bilin by zinc-induced fluorescence (right panel). A slight size difference in the 1-908 constructions was evident, because the 6His-TEV tag was not removed from the PΦB-containing biliprotein prior to electrophoresis.

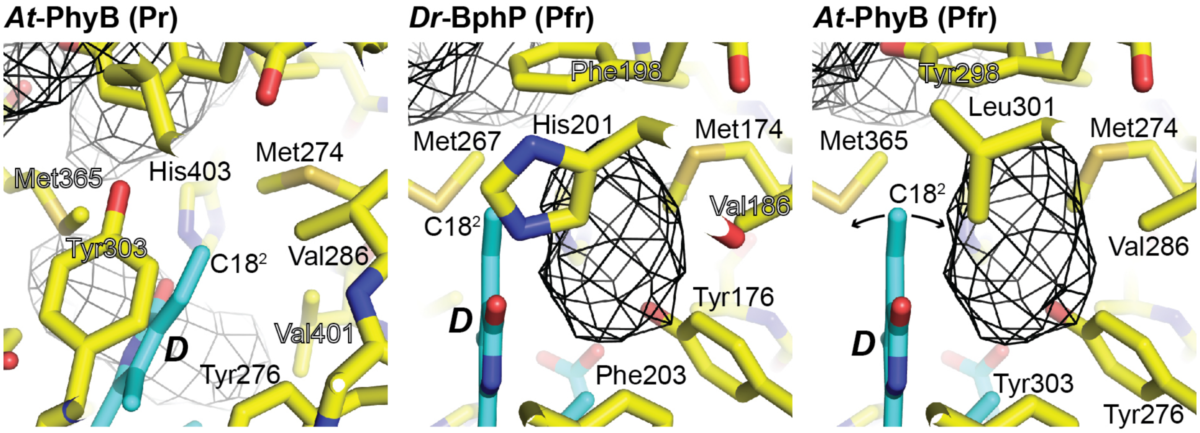

Supplementary Figure S5. Modeling the PhyB binding-pocket predicts that conformational freedom of the C18 ethyl group in PCB could enhance stability of the Pfr state. All three models replaced the native bilin with PCB. (Left) The Pr state of Arabidopsis PhyB was analyzed using the PSM structure described by Burgie et al. (PDB ID: 4OUR)[21]. Here, no significant open pocket was observed near $C18^2$ of PCB. (Middle) The Pfr state of *D. radiodurans* (*Dr*)-BphP was analyzed using the PSM structure described by Burgie et al. (PDB ID: 5C5K)[14]. A salient hydrophobic pocket indicated by a van der Waals surface (black mesh) was visible near $C18^2$ of the D-ring. (Right) The predicted bilin-binding pocket of Arabidopsis PhyB using the model for the Pfr state of *Dr*-BphP as a guide. The same hydrophobic pocket was again visible near $C18^2$ of the D-ring. Assuming flexibility of the C18 ethyl moiety in PCB, rotation (arrows) could encourage a more intimate contact of the D pyrrole ring into the GAF domain cavity. The black mesh at the top left of each panel indicates an external van der Waals surface of the respective Phy. For ease of viewing, the amino acid and bilin carbons were colored yellow and cyan, respectively.
